# Non-tuberculous mycobacteria (NTM) in Zambia: prevalence, clinical, radiological and microbiological characteristics

**DOI:** 10.1186/s12879-015-1264-6

**Published:** 2015-11-06

**Authors:** Pascalina Chanda-Kapata, Nathan Kapata, Eveline Klinkenberg, Lutinala Mulenga, Mathias Tembo, Patrick Katemangwe, Veronica Sunkutu, Peter Mwaba, Martin P. Grobusch

**Affiliations:** Department of Disease Surveillance, Control and Research, Ministry of Health, Lusaka, Zambia; Center of Tropical Medicine and Travel Medicine, Amsterdam Medical Centre, University of Amsterdam, Amsterdam, Netherlands; Department of Epidemics and Disease Control, Ministry of Community Development, Mother and Child Health, Lusaka, 10101 Zambia; KNCV Tuberculosis Foundation, The Hague, Netherlands; Department of Global Health and Development, Amsterdam Medical Centre, Amsterdam, Netherlands; Department of Clinical Care and Diagnostics, Chest Diseases Laboratory, Ministry of Health, Lusaka, 10101 Zambia; Tuberculosis Laboratory, Tropical Diseases Research Centre, Ndola, Zambia; Tuberculosis Laboratory, University Teaching Hospital, Lusaka, 10101 Zambia; Radiology Department, University Teaching Hospital, Lusaka, 10101 Zambia

**Keywords:** Epidemiology, Survey, Non-tuberculous mycobacteria, Emerging, Public health, Diagnosis

## Abstract

**Background:**

Non-tuberculous mycobacteria (NTM) infection is an emerging health problem. We present here the Zambia-specific national level data of prevalence, symptomatic, radiological and microbiological characteristics of NTM, using results from a national Tuberculosis (TB) prevalence survey.

**Methods:**

This was a cross-sectional study of the prevalence of NTM among adults aged 15 years and above, who were participants in a national TB prevalence survey. Participants who had either an abnormal chest x-ray or were symptomatic were considered presumptive TB cases and submitted sputum for smear and culture analysis. HIV testing was performed on an opt-out basis. Symptomatic NTM prevalence was estimated from individual level analysis.

**Results:**

Of the 6,123 individuals with presumptive TB, 923 (15.1 %) were found to have NTM, 13 (0.2 %) were MTB/NTM co-infected and 338 (5.5 %) were contaminated (indeterminate). The prevalence of symptomatic NTM was found to be 1,477/100,000 [95 % CI 1010–1943]. Smear positivity, history of cough or chest pain and HIV positivity were risk factors for NTM.

**Conclusion:**

This first study to estimate the national prevalence of NTM in Zambia indicates that the burden is high. The NTM occurrence in Zambia constitutes both a public health and ethical issue requiring action from health managers.

## Background

Non-tuberculosis Mycobacteria (NTM) refer to all *Mycobacterium* species other than the pathogens of the *M.tuberculosis* complex (MTBC), *M. leprae* and M. *ulcerans,* and rarely cause disease in humans. However, they may cause disease in individuals with pre-existing lung disease or immunodeficiency while some species may cause disease in elderly women without underlying pulmonary disease or immunodeficiency [1, 2] NTM are commonly found in the environment and can be isolated from sources such as soil, milk, dust, animals and birds [1]. Over 140 species of NTM have been reported in literature with the majority of these species rarely being isolated in clinical samples [3]. The most common group of NTM is the *Mycobacterium avium* complex (MAC) comprising the clinically important members *M. intracellulare M. avium and M.chimaera*. Other potentially pathogenic NTM species include *M. chelonae*, *M. kansasii*, *M. xenopi, M. marinum, M. abscessus and M. fortuitum* [4–6].

Among the diseases caused by NTM, pulmonary disease is the most frequent, followed by lymphadenitis in children, skin disease caused by *Mycobacterium marinum* (especially common in fish tank owners) and other extra-pulmonary or disseminated infections [3, 5, 7]. NTM can exhibit clinical and radiographic features that are similar to those by MTBC [8, 9] especially in persons with progressed cellular immune dysfunction. Although this is not the case in all studies, in Brazil, Dos Santos and colleagues [10] compared radiological features between MTBC and NTM and found some differences in patients with AIDS. Species differentiation is needed to tell NTMs safely apart from MTBC in individuals suggestive of having TB [11]. However, distinguishing between MTBC and NTM diseases may be a challenge, especially in low resource settings where microscopy is the mainstay for MTBC diagnosis. Although microscopy for acid-fast bacilli (AFB) allows for rapid diagnosis of mycobacteria, it does not, however, differentiate *M. tuberculosis* from NTM. A recent study in Nigeria [12] showed that failure to characterize AFB positive NTM lung infections has led to misclassification and incorrect treatment as pulmonary tuberculosis since all diagnosed sputum smear positive patients are placed on TB treatment. In order to differentiate the two, traditional culture methods may be required but these may take up to 6 weeks [13].

There is limited literature available on the extent of the burden of pulmonary disease due to NTM in sub-Saharan Africa. Studies conducted in the later part of the 1950s and early 1960s [14] identifying mycobacterial groups using traditional tools like characteristics such as growth rate and morphology reported the isolation of *NTM* from both tuberculosis patients and the general public in some African countries, Zambia included. The study as a whole found that the frequency of NTM strains differed; 19.8 % NTM cases were identified among 657 cultures isolated from specimens of a general population survey, whereas only 1.1 % NTM cases were identified from 7,580 cultures from tuberculosis patients. Studies conducted in Nigeria and Tanzania suggest NTM infections could be associated with HIV co-infection [15–17]. A prospective cohort study among HIV positive patients in Cote d’Ivoire reported a 10 times higher incidence of NTM infection among patients with baseline CD4 cell counts below 100 cells/mm^3^ [18].

In Zambia, clinically relevant infection due to NTM was shown to occur in both HIV-positive and HIV-negative patients in a rural hospital [19]. In another study, Buijtels and colleagues [20] evaluated the clinical relevance of NTM isolated from chronically ill patients and healthy controls in Zambia and found that the proportion of NTM-positive sputum samples was significantly higher in the patient group (11 %) than in controls (6 %). The most frequently isolated NTM was *M. avium* complex. Unlike HIV, sex and age; being underweight and consumption of tap water were identified as risk factors for NTM infection.

While it is acknowledged that the prevalence and the species of NTM isolated is increasing [5, 7, 8], the clinical relevance and characteristics of the NTMs in different settings remain to be established.

We present here the Zambia-specific data on national level prevalence, symptomatic, radiological and microbiological characteristics of NTMs, using results from a national Tuberculosis prevalence survey. To our knowledge, this is the first large data set to explore the prevalence of NTM from a national representative sample covering all provinces and to discuss radiological characteristics of NTM in Zambia.

## Methods

This was a cross-sectional study of the prevalence of NTM among adults aged 15 years and above, who were participants in a national TB prevalence survey. All individuals with an abnormal chest x-ray or positive symptom screening result (i.e. cough or chest pain, or fever lasting more than 2 weeks or more) were requested to submit two sputum samples; one on the spot and another in the morning. The samples were then transported in cooler boxes packed with cold gel within 24 h of collection to each of the three central reference laboratories (CRLs) for processing. At the laboratories, the sputum samples were first decontaminated in 1 % sodium dydroxide (NaOH) working solution for 15 min and then stained for AFB using the Auramine-Phenol method. The smears were graded as smear positive for mycobacterial infection using both qualitative and quantitative grading methods. Thereafter, the samples were cultured using liquid media by mycobacteria growth indicator tube (MGIT) (BD BACTEC^TM^). Mycobacterial growth was detected and identified as either MTB or NTM using the capilia (TAUNS) method. The laboratory team was blinded to the radiology or symptom status of the study participants; they only had a Personal Identification Number (PIN) and sample.

Before collection of sputum samples; each symptomatic survey participant also underwent an in-depth questionnaire. Initial reading of chest radiographs was done by medical officers in the field and classified as either normal or abnormal. All images subsequently underwent reading by expert radiologists who classified the images using a pre-defined criteria [21] as follows:

- N = Normal
- AD - NS = Abnormality detected - not significant
- ADS-NA = Abnormality detected, significant no active disease
- ADS-NTB = Abnormality detected, significant not-TB
- ADS-TB = Abnormality detected, significant TB

All the abnormal ‘not TB’ images were then re-read to further specify the abnormality so as to make a radiological diagnosis. The central reading by radiologists was performed independent of the microbiology results.

The HIV status for each of the NTM cases was established using the HIV testing results which were obtained using a separate consent during field level HIV screening of survey participants. The screening test used was Determine® HIV-1/2 (Inverness Medical Japan, Co, Ltd) and all positives were further confirmed by Unigold® HIV-1/2 (Trinity Biotech, Wicklow, Ireland). Discordant results (positive on Determine® but negative on Unigold®) were recorded as indeterminate.

At the central data management unit, the results of the symptom screening, chest x-ray screening and laboratory results were merged to create one final database for analysis. The files were merged using PINs which were unique to each participant in the survey. This was done to ensure that for each NTM, data on radiology, clinical history and microbiology was complete. Only the data manager had access to the complete merged data set. This was done to ensure confidentiality but also to reduce bias during case detection or diagnosis.

Data analysis was performed using STATA version 12. Means and frequencies were generated taking into account of the Random Standard Errors using the default “*svy*” command during analysis.

Chi-square tests were performed to establish relationship between the categorical or binary variables. Linear regression analysis was done to show the association between NTM and different exposure variables such as HIV status, smear positivity, history of cough or chest pain or fever.

The prevalence of NTM was estimated using cluster level and individual level analysis as recommended by Floyd et al. [22].

The study protocol was cleared by the University of Zambia Biomedical Research Ethics Committee (UNZABREC) No: 020-08-12. Authorisation to conduct the survey was sought in line with the existing national policies and guidelines at national, provincial and district levels. Written informed consent was obtained from all individuals who agreed to participate in the survey.

## Results

Of the 6,123 with presumptive MTB, 923 (15.1 %) were found to have NTM, 265 (4.3 %) were MTB, 13 (0.2 %) were MTB/NTM co-infected and 338 (5.5 %) were contaminated (indeterminate). Basic cohort characteristics are shown in Table 1. Most of the participants with NTM were from the lowest-wealth quintile. 71 % of the NTM infected participants were symptomatic, either having a cough and/or chest pain and/or fever for two weeks or more. 52 % of NTM infected participants had an abnormal field chest x-ray reading. 23 % (210) of the participants with NTM were both symptomatic and had an abnormal CXR reading at field level. Chest x-ray results were not available for 2/923 participants with NTM.

**Table 1:** Background characteristics of the survey participants with NTM

Variable	Number	Percent (%)
Sex (*N* = 923)		
Male	446	48.32
Female	477	51.68
Setting (*N* = 923)		
Rural residence	708	76.71
Urban residence	215	23.29
Age group (*N* = 923)		
15–24	80	8.67
25–34	128	13.87
35–44	154	16.68
45–54	143	15.49
55–64	154	16.68
65+	264	28.60
Education (*N* = 923)		
No schooling	190	20.59
Primary	493	53.41
Secondary	226	24.49
Tertiary	14	1.52
Wealth Quintile (*N* = 834)		
Lowest	215	25.78
Second Lowest	208	24.94
Middle	197	23.62
Fourth	108	12.95
Highest	106	12.71
Symptom Screening (*N* = 923)		
Postive	655	70.96
Negative	268	29.04
Field chest x-ray (*N* = 923)		
Normal	443	48.00
Abnormal	478	51.79
Not done	2	0.22

Some of the abnormal chest radiographs showed evidence of bronchiectasis, nodules, fibrosis or cavitation as shown in Table 2. Majority of the participants with NTM had negative smear (94.5 %) and 62 % were HIV negative.

**Table 2:** Clinical, radiological and microbiological characteristics of individuals with NTM

Clinical Symptoms:	Number	Percent (%)
Cough	489	52.98
Fever	324	35.10
Chest pain	656	71.07
Unintentional weight loss	329	35.64
Expert radiology reading:		
Bronchiectasis	66	7.15
Nodules	203	21.99
Cavitation	127	13.76
Fibrosis	140	15.17
Miliary TB	19	2.06
Not available	368	39.87
Smear:		
Positive	51	5.53
Negative	872	94.47
HIV status:		
Positive	54	5.85
Negative	572	61.97
Not available	297	32.18

Comparing the three cardinal symptoms collected in the survey (Table 3); 277 individuals with NTM had no history of either cough or chest pain or fever and 52 reported a history of fever only without any of the other two (chest pain and cough). A total of 423 individuals reported having all the three symptoms.

**Table 3:** Comparison of the occurrence of symptoms among individuals with NTM (Absolute numbers)

	NTM individual with fever and NTM individual with chest pain			
Cough	No		Yes	
	No	Yes	No	Yes
No	277	394	52	181
Yes	138	353	41	423

The risk factors of NTM included smear positivity; history of cough or chest pain or fever or weight loss and being HIV positive as shown in Table 4.

**Table 4:** Risk factors of Non-tuberculous mycobacteria

Variable	Number (%)	Odds ratio [95 % CI]	*p*-value
Smear positive	51 (5.53)	2.01 [1.82–2.21]	0.000
Cough	489 (52.98)	0.804 [0.723–0.895]	0.000
Chest pain	656 (71.07)	1.25 [1.11–1.40]	0.000
Fever at night	324 (35.10)	1.13 [1.01–1.27]	0.027
Unintentional weight loss	329 (35.64)	0.855 [0.763–0.953]	0.007
HIV positive	54 (5.85)	0.941 [0.913–0.970]	0.000

Prevalence of symptomatic NTM among TB survey participants aged 15 years and above was found to be 1,477/100,000 [95 % CI 1010–1943]. Symptomatic NTM prevalence was found to be significantly higher among the rural than urban participants (1927 vs 642/100,000 population). However, there was no significant difference in the prevalence of symptomatic NTM by sex (1,577/100,000 versus 1403/100,000). Among the provinces, NTM prevalence was highest in Western (9,824/100,000) and lowest in Copperbelt (309/100,000) (Table 5). The participation rate for Western province was low hence this may have resulted into the wide confidence interval of the prevalence estimate.

**Table 5:** Symptomatic Non-tuberculous mycobacteria prevalence per 100,000 adult population

Variable	Prevalence estimate	95 % Confidence interval
Overall	1477	1010–1943
Setting		
Rural	1927	1244–2611
Urban	642	332–951
Sex		
Male	1577	1140–2014
Female	1403	885–1922
Age group (years)		
15–24	452	268–639
25–34	1010	649–1371
35–44	1394	943–1844
45–54	2038	1247–2829
55–64	2803	1575–4032
65+	5160	3619–6702
Age group (years) and sex	Male	Female
15–34	712 (447–976)	661 (392–930)
35–54	1911 (1363–2460)	1778 (954–2601)
55+	4124 (2822–5426)	3841 (1975–5707)
Wealth quintile		
Lowest	2582	1628–3535
Second lowest	2638	1635–3640
Middle	1773	1121–2424
Fourth	778	359–1196
Highest	464	285–643
Education level		
No schooling	3495	2010–4980
Primary school	1858	1301–2415
Secondary school	752	512–992
Tertiary education	339	19–658
Bacteriological Tuberculosis status		
Negative	1468	996–1940
Positive	6773	3453–10093
Smear Tuberculosis status		
Negative	1479	1007–1951
Positive	8527	3180–13874
HIV status		
HIV negative	1549	1008–2090
HIV positive	2187	1428–2946
HIV status unknown	1249	863–1635
By Province		
Central	3222	739–5706
Copperbelt	309	161–458
Eastern	695	411–979
Luapula	2150	524–3777
Lusaka	729	395–1062
Muchinga	3794	1818–5771
Northern	1217	650–1784
North Western	1688	1077–2299
Southern	822	294–1349
Western	9824	3767–15881

The prevalence of symptomatic NTM increased with participant’s age from 452/100,000 among those aged 15–24 years to 5160/100,000 among those aged 65 years and above. The symptomatic NTM prevalence was highest among the lower wealth quintiles and participants without schooling. Participants with bacteriologically confirmed MTB had significantly higher symptomatic NTM prevalence than those without (6773 vs 1468/100,000). Participants with smear positive TB had a higher prevalence of symptomatic NTM than those with a negative smear (8527 vs 1479/100,000). Considering HIV status, the HIV positive participants had higher symptomatic NTM prevalence than the HIV negative counterparts (2187 vs 1549/100,000) but the difference was not significant.

## Discussion

This study represents a first attempt to quantify NTM prevalence nation-wide in Zambia. The focus was on individuals with NTM coupled with both symptom and radiological profiles. The high prevalence of NTM among symptomatic participants found in this population based study demonstrates that NTM is a public health problem. There is a need to further identify the species and come up with case management guidelines in order to appropriately manage the people in whom NTM is causing illness. Symptomatic NTM prevalence was found to be approximately two times higher than MTB and 14 % of the smear positives were found to be NTM [23]. Therefore, NTM patients could potentially be mismanaged as MTB. Besides a public health issue this is also an ethical issue requiring no further neglect. Although the World Health Organization (WHO) has described Neglected Tropical Diseases (NTDs), this list conspicuously does not yet include any of the NTM.

As smears are routinely used to diagnose TB and to initiate treatment, it could be that symptomatic NTM cases seeking care in health facilities are misdiagnosed and placed on TB treatment. Without adequate clinical response, these cases may subsequently be misdiagnosed as TB treatment failures. Thus, false MTB-positives may in fact be clinical NTM and if the treatment outcomes do not improve; the patient may mistakenly be classified as MDR-TB. Such patient mismanagement may negatively impact the health status of the individual and consequently pose additional cost to the health system. Therefore, appropriate NTM case management is required in order to effectively and efficiently manage the MTB problem.

The radiological abnormalities for NTM observed in this survey are consistent with what has been reported elsewhere [5]. The pulmonary presentation of NTM was characterized by the presence of bronchiectasis; cavitation; pneumoconiosis, fibrosis and nodules. The cardinal symptoms of fever, chest pain and unintentional weight loss were prevalent in the survey participants with NTM. This may suggest that NTM in Zambia is causing symptomatic disease.

The higher NTM prevalence among HIV positive individuals brings a further challenge of managing NTM/HIV and its associated side effects for potential patients. Even though, the burden of NTM was higher among the HIV positive, there is also a substantial burden among the HIV negative that cannot be ignored.

The NTM prevalence in Zambia seems to increase with age. This was true for both male and female supporting earlier evidence from Greece and the United States of America [5, 24, 25]. Nonetheless, it will be important to establish if the species causing disease are occurring in similar proportions among the male and females since reported symptoms were found to differ by gender.

Studies from Nigeria, Tanzania and South Africa have shown that NTMs may be occurring among patients seeking care for MTB [15, 16, 26] with varying frequency by geographical locations [27]. The NTM prevalence isolated in this Zambia survey is higher than what was found in Oregon in the United States of America and Ontario in Canada [28, 29]. However, the differences in the study population and analytical approach partially contribute to the variations. Our study also suggests potential interaction or misdiagnosis with MTB as in the American and Canada studies. Therefore, a comprehensive multi-country evaluation of NTM is needed to better understand the extent of the NTM burden on the globe and design strategic action.

Given the dimension of the problem and the consequences for the individual patient as well as the health system in terms of potential to cause negative impact on treatment cost and duration, and possible spread of the organism, speciation and resistance testing may be cost-effective in the long term in order to enable correct and timely treatment of patients.

A potential limitation is that the study was conducted within the national TB prevalence survey design, as a result, we are unable to fully apply the existing NTM disease classification criteria because the methods used for sputum collection were with the primary purpose of estimating MTB prevalence. However, the availability of samples from various subnational levels provides opportunity for better estimation of the NTM burden and provides geographic guidance for potential programmatic focus.

This study is the first attempt to estimate the national burden of NTM in Zambia. Further studies are recommended to fully understand causal and etiological pathways.

## Conclusions

Our data confirm that the problem of NTM is substantial in Zambia. Synopsis of epidemiological, clinical and radiological information is needed to support an informed decision on whether a patient has disease resulting from MTBC or NTM infection. Rigorous surveillance strategies are required to curb the further spread of this neglected mycobacterial disease.

## References

[1] Jamison DT (2006). Disease and Mortality in Sub-Saharan Africa.

[2] Field SK, Fisher D, Cowie RL (2004). Mycobacterium avium complex pulmonary disease in patients without HIV infection. Chest.

[3] van Ingen J (2013). Diagnosis of nontuberculous mycobacterial infections. Semin Respir Crit Care Med.

[4] Falkinham JO (1996). Epidemiology of infection by nontuberculous mycobacteria. Clin Microbiol Rev.

[5] Griffith DE, Aksamit T, Brown-Elliott BA, Catanzaro A, Daley C, Gordin F (2007). An official ATS/IDSA statement: diagnosis, treatment, and prevention of nontuberculous mycobacterial diseases. Am J Respir Crit Care Med.

[6] Johnson MM, Odell JA (2014). Non tuberculous mycobacterial pulmonary infections. J Thorac Dis.

[7] Aksamit TR, Philley JV, Griffith DE (2013). Nontuberculous mycobacterial (NTM) lung disease: The top ten essentials. Respir Med.

[8] Satyanarayana G, Heysell KS, Scully KW, Houpt ER (2011). Mycobacterial infections in a large Virginia hospital, 2001–2009. BMC Infect Dis.

[9] Matveychuk A, Fuks L, Priess R, Hahim I, Shitrit D (2012). Clinical and radiological features of *Mycobacterium kansasii* and other NTM infections. Respir Med.

[10] dos Santos RP, Scheid KL, Willers DM, Goldani LZ (2008). Comparative radiological features of disseminated disease due to Mycobacterium tuberculosis vs non-tuberculosis mycobacteria among AIDS patients in Brazil. BMC Infect Dis.

[11] Taiwo B, Glassroth J (2010). Nontuberculous mycobacterial lung diseases. Infect Dis Clin North Am.

[12] Pokam BT and Asuquo AE: Acid-Fast Bacilli Other than Mycobacteria in Tuberculosis Patients Receiving Directly Observed Therapy Short Course in Cross River State, Nigeria. *Tuberculosis Research and Treatment* 2012, Article ID 301056, 4 pages, 2012. doi:10.1155/2012/301056. 10.1155/2012/301056 PMC3413975 22919477

[13] Cook VJ, Turenne CY, Wolfe J, Pauls R, Kabani A (2003). Conventional methods versus 16S ribosomal DNA sequencing for identification of non-tuberculous mycobacteria: Cost analysis. J Clin Microbiol.

[14] Zykov MP, Roulet H, Gaya N (1967). Non-tuberculosis mycobacteria in Africa: Isolation and identification. Bull World Health Organ.

[15] Aliyu G, El-Kamary S, Abimiku A, Brown C, Tracy K, Hungerford L (2013). Prevalence of Non-Tuberculous Mycobacterial Infections among Tuberculosis Suspects in Nigeria. PLoS One.

[16] Crump AJ, Ramadhani HO, Morrissey BA, Saganda W, Mwako MS, Yang L (2009). Invasive disease caused by nontuberculous mycobacteria. Tanzania Emerg Infect Dis.

[17] Henkle E, Winthrop KL (2015). Nontuberculous mycobacteria infections in immunosuppressed hosts. Clin Chest Med.

[18] Bonard D, Messou E, Seyler C, Vincent V, Gabillard D, Anglaret X (2004). High incidence of atypical mycobacteriosis in African HIV-infected adults with low CD4 cell counts: a 6-year cohort study in Cote d’Ivoire. AIDS.

[19] Buijtels PC, van der Sande MA, Parkinson S, Verbrugh HA, Petit P, van Soolingen D (2010). Isolation of non-tuberculous mycobacteria at three rural settings in Zambia; a pilot study. Clin Microbiol Infect.

[20] Buijtels PC, van der Sande MA, de Graaff CS, Parkinson S, Verbrugh HA, Petit PLC (2009). Non-tuberculous mycobacteria. Zambia Emerg Infect Dis.

[21] World Health Organisation. Tuberculosis Prevalence Surveys: a handbook. WHO/HTM/TB/201.17. WHO, Geneva, Switzerland. Available [Online] from: http://www.who.int/tb/advisory_bodies/impact_measurement_taskforce/resources_documents/limebook_appendix/en/

[22] Floyd S, Sismandis C, Yamada N, Daniel R, Lagahid J, Mecatti F (2013). Analysis of tuberculosis prevalence surveys: new guidance on best-practice methods. Emerging themes in epidemiology.

[23] Government of The Republic of Zambia. National Tuberculosis Prevalence Survey 2013–2014. Ministry of Health. Lusaka, Zambia. 2015

[24] Panagiotou M, Papaioannou AI, Kostikas K, Paraskeua M, Velentza E, Maria Kanellopoulou M, Filaditaki V, and Karagiannidis N: The Epidemiology of Pulmonary Nontuberculous Mycobacteria: Data from a General Hospital in Athens, Greece, 2007–2013. *Pulmonary Medicine 2014*, Article ID 894976, 9 pages. doi:10.1155/2014/894976 10.1155/2014/894976 PMC4123541 25132991

[25] Mirsaeidi M, Machado RF, Garcia JGN, Schraufnagel DE (2014). Nontuberculous mycobacterial disease mortality in the United States, 1999–2010: a population-based comparative study. PLoS One.

[26] Sookan L, Coovadia YM (2014). A laboratory-based study to identify and speciate non-tuberculous mycobacteria isolated from specimens submitted to a central tuberculosis laboratory from throughout KwaZulu-Natal Province. South Africa South African Medical Journal.

[27] Gunaydin M, Keramettin Y, Eroglu C, Sanic A, Ceyhan I, Erturan Z (2013). Distribution of nontuberculous mycobacteria strains. Ann Clin Microbiol Antimicrob.

[28] Kendall BA, Varley CD, Hedberg K, Cassidy PM, Winthrop KL (2010). Isolation of non-tuberculous mycobacteria from the sputum of patients with active tuberculosis. Int J Tuberc Lung Dis.

[29] Marras TK, Chedore P, Ying AM, Jamieson F (2007). Isolation prevalence of pulmonary non-tuberculous mycobacteria in Ontario, 1997 2003. Thorax.

